# Multi-Layer Feature Based Shoeprint Verification Algorithm for Camera Sensor Images

**DOI:** 10.3390/s19112491

**Published:** 2019-05-31

**Authors:** Xinnian Wang, Yanjun Wu, Tao Zhang

**Affiliations:** 1 School of Information Science and Technology, Dalian Maritime University, Dalian 116026, China; wuyanjun@dlmu.edu.cn; 2 School of Physics and Electronics Technology, Liaoning Normal University, Dalian 116026, China; lnnuzt@lnnu.edu.cn

**Keywords:** shoeprint verification, shoeprint partition model, multi-layer feature, forensic sciences, shoeprint matching strategy

## Abstract

As a kind of forensic evidence, shoeprints have been treated as important as fingerprint and DNA evidence in forensic investigations. Shoeprint verification is used to determine whether two shoeprints could, or could not, have been made by the same shoe. Successful shoeprint verification has tremendous evidentiary value, and the result can link a suspect to a crime, or even link crime scenes to each other. In forensic practice, shoeprint verification is manually performed by forensic experts; however, it is too dependent on experts’ experience. This is a meaningful and challenging problem, and there are few attempts to tackle it in the literatures. In this paper, we propose a multi-layer feature-based method to conduct shoeprint verification automatically. Firstly, we extracted multi-layer features; and then conducted multi-layer feature matching and calculated the total similarity score. Finally, we drew a verification conclusion according to the total similarity score. We conducted extensive experiments to evaluate the effectiveness of the proposed method on two shoeprint datasets. Experimental results showed that the proposed method achieved good performance with an equal error rate (EER) of 3.2% on the MUES-SV1KR2R dataset and an EER of 10.9% on the MUES-SV2HS2S dataset.

## 1. Introduction

With the popularity of crime investigation dramas and novels, more and more people know what investigators are looking for while solving a case. This kind of knowledge has affected the investigation and solving of cases, because some criminals may conceal their crime traces after they commit crimes. In fact, if there are no other forms of criminal evidence, as the only remaining evidence at a crime scene, shoeprint may very well reveal some available clues to a particular case.

Shoeprint conveys many important human characteristics, such as walking habits and identity, and it may play a vital role in crime investigation. The typical uses of shoeprints are to link cases, to testify whether the shoeprints are left by the same shoe, then identify the suspect. It is clear that some typical forensic evidence (fingerprints and palmprints) can be successful in linking evidence to a suspect for the evidence’s uniqueness. Similar with the evidence stated previously, shoeprint evidence has its own unique identifying characteristics. The identifying characteristics can be classified into three categories: class characteristics, wear characteristics, and individual identifying characteristics [1]. Class characteristics are shoe patterns that are usually created in the manufacturing process, and different shoes may have different shoe patterns. Class characteristics are usually used to recognize or retrieve shoeprints with similar shoe patterns. Wear characteristics are those changes on the outsoles that are caused by natural erosion or wear of the shoe which can reflect personal walking habits [1]. Wear characteristics of shoes worn by different persons are different. Individual identifying characteristics refer to random tears, cuts, and punctures on the outsoles, which are produced in the process of human wearing. Individual identifying characteristics may make shoes with the same class characteristics different. These characteristics are usually applied for shoeprint verification, which is to determine whether two shoeprints could, or could not, have been made by the same shoe or person.

Most of the researches on shoeprint tend to concentrate on shoeprint classification and retrieval by using class characteristics. Rida et al. [2] provide an overview of the research carried out in forensic shoeprint retrieval. According to the scope of representation, shoeprint classification and retrieval methods may roughly fall into three categories: global features-based methods, region features-based methods, and interest point-based methods. Methods using the global features usually take into consideration the whole shoeprint for extracting features, for example, the moments invariant methods [3,4], frequency domain methods [5,6,7,8,9,10], and convolutional neural network-based method [11,12]. Methods using the region feature usually divide shoeprints into different regions and extract features from these regions, for example, the maximally stable extreme regions (MSER)-based methods [13,14], the periodic pattern-based methods [15], the compositional active basis model-based methods [16,17], and the sparse representation methods [18]. Interest point-based methods detect interest points and use these points to recognize shoeprints (e.g., [19,20,21]). Luostarinen et al. [22] evaluated different shoeprint retrieval methods and compared their performance under shoeprints with different quality conditions. Their results show that methods based on local interest points with RANSAC and Fourier–Mellin transform can achieve good retrieval performance under different quality conditions. Although the above methods can achieve a good performance on shoeprint retrieval, they may fail to handle shoeprint verification. Class characteristics of a shoe can provide informative information to recognize shoe patterns but are not unique enough to verify two shoeprints.

In forensic practice, shoeprint verification is manually performed by forensic experts. Forensic experts first manually label the individual identifying characteristics, then compare them in their own opinions; and finally draw a conclusion as to whether the two shoeprints could have come from the same shoe according to their own opinions. This kind of comparison method is too dependent on experts’ experience, and the conclusions of different experts are not always similar.

Yekutieli et al. [23] proposed a system to compare individual identifying characteristics with a physical matching method. The individual identifying characteristics used in their method are first marked manually, and then the contour of each individual identifying characteristic is extracted for comparison. This method cannot perform shoeprint verification in an automatic manner. To the best of our knowledge, there are no works focusing on automatic shoeprint verification. Therefore, in this paper, we focus on an automatic shoeprint verification algorithm.

The main contributions of the proposed method are summarized as follows:

(i) we propose a multi-layer feature-based shoeprint verification algorithm that can be used in forensic practice;
(ii) we introduce a shoeprint partition model (SPM) to analyze shoeprints, which considers foot anatomy and the relationship between shoe and foot, and facilitates analyzing shoeprints accurately in practice;
(iii) we propose an individual identifying characteristics detection method to perform characteristics detection automatically;
(iv) we propose a shoeprint image matching strategy. The shoeprint is divided into nineteen sections. Similarities of each section are computed respectively, and the total similarity between the two images is a weighted sum.

The rest of this paper is organized as follows: Section 2 describes the shoeprint acquisitions and datasets, Section 3 details the proposed method, Section 4 provides the performance experiments and the detailed evaluation of the proposed method and Section 5 concludes the paper.

## 2. Shoeprint Acquisitions and Datasets

**Figure 1:**
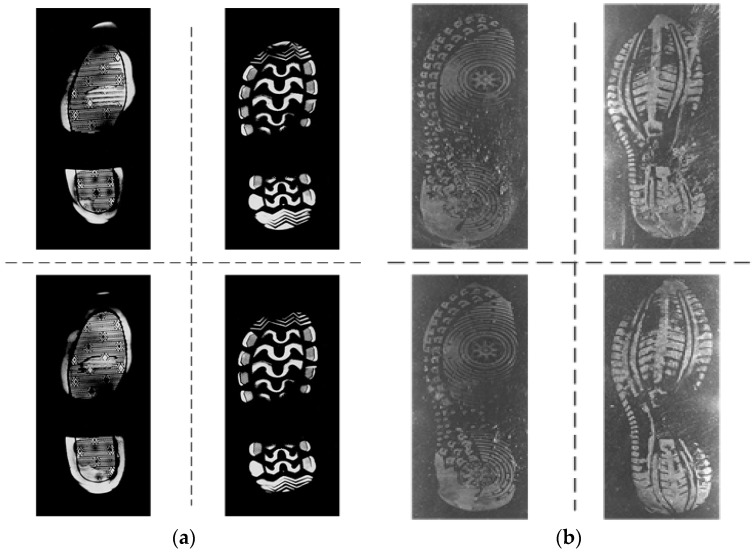
Sample of shoeprints in the datasets. (**a**) Samples of shoeprints in the MUES-SV1KR2R dataset; (**b**) Samples of shoeprints in the MUES-SV2HS2S dataset.

To testify the effectiveness of the proposed methods, we build two kinds of shoeprint verification datasets named MUES-SV1KR2R and MUES-SV2HS2S. Samples from the two shoeprint image datasets are shown in Figure 1. 

**Figure 2:**
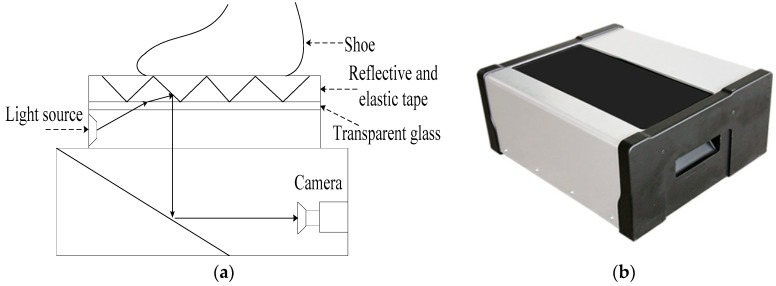
Illustration of the reference shoeprint scanner. (**a**) Structural diagram of the reference shoeprint scanner; (**b**) the real system of the reference shoeprint scanner.

Images in MUES-SV1KR2R dataset are pressure images acquired by using the shoeprint scanners, and the imaging process is shown in Figure 2. When one steps on the reflective tape (the height of the reflective tape is about 1 mm), different height downward bulges are formed on the surface of the reflective tape because of the uneven pressures. The higher the pressure is, the higher the downward bulges are. The lights of the light source shine through the transparent glass and on the surface of these downward bulges. The lights then produce shadowing between the different heights area of those downward bulges and the lights are reflected to the camera by the mirror. Different amounts of light are captured by the camera for the different heights of bulges, and a shoeprint image can be scanned at 300 dpi. Then the obtained shoeprint images are stored in the MUES-SV1KR2R dataset. Some samples in the MUES-SV1KR2R dataset are shown in Figure 1a. The MUES-SV1KR2R dataset consists of more than 1200 pairs of shoeprint images, and each pair of shoeprints has the same shoe patterns. Of 1200 shoeprint pairs, 600 pairs of shoeprints are made by the same shoes. We refer this kind of shoeprint as a reference or suspect shoeprint. Images in the dataset are registered and have the same spatial resolution. Most images in the database are clear and full. Shoeprint images are available as 3600 by 1800 pixels. 

**Figure 3:**
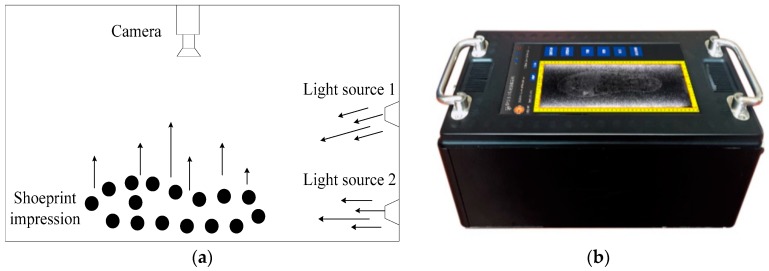
Illustration of the crime scene shoeprint scanner. (**a**) Structural diagram of the crime scene shoeprint scanner; (**b**) the real system of the crime scene shoeprint scanner.

Images in MUES-SV2HS2S are crime scene shoeprints collected by using the crime scene shoeprint scanners, and the imaging process is shown in Figure 3. For an impression left on the ground, the oblique lights from the light source shine to create shadows between the low and high areas of the impression and provide a great amount of contrast between those areas. Then the contours and contents within the impression can be revealed and captured by the camera. For impressions in sand, soil, or snow, the oblique light from the light source 1 can provide the best contrast, and for those impressions in dust or residue, the oblique light from light source 2 is most effective at a very low angle. The distance from light source 1 to the shoeprint is about 5 cm. A shoeprint image is scanned at 300 dpi according to the above process, and the shoeprint images are stored in the MUES-SV2HS2S dataset. Some samples in the MUES-SV2HS2S dataset are shown in Figure 1b. The MUES-SV2HS2S dataset consists of more than 256 pairs of shoeprints with same shoe patterns. Of 256 shoeprint pairs, 128 pairs of shoeprints are made by the same shoes. We refer this kind of shoeprint as a crime scene shoeprint. Images in this dataset are registered and have the same spatial resolution. Shoeprint images are available as 3600 by 1800 pixels.

## 3. Methods

**Figure 4:**
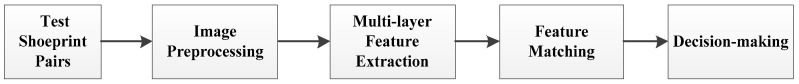
Framework of the proposed shoeprint verification system.

The proposed shoeprint verification scheme consists of four basic modules: shoeprint preprocessing module, multi-layer feature extraction module, multi-layer feature matching module, and decision-making module, as shown in Figure 4. Each of these modules plays a vital role in the shoeprint verification system. The functionalities of these modules are stated as that: (1) preprocessing module aims at separating the valid shoeprints from the complex backgrounds, and conducting shoeprint registration and partition; (2) multi-layer feature extraction module aims at representing the characteristics of shoeprints on global layer, partial layer and individual identifying layer; (3) multi-layer feature matching module compares the multi-layer features; and (4) decision-making module calculates the total similarity score, and then makes the decision that the two shoeprints are left by the same shoe or not.

### 3.1. Shoeprint Preprocessing

**Figure 5:**
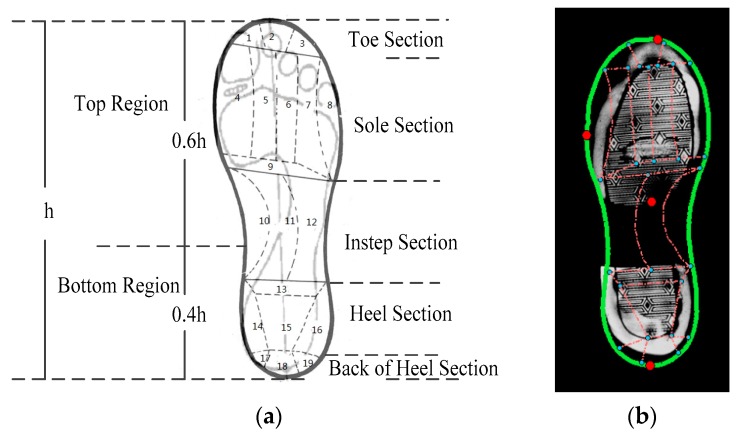
Illustration of the shoeprint partition model. (**a**) The layout of 19 subsections of a shoeprint; (**b**) Example of the Shoeprint Partition Model (SPM) for a full shoeprint.

This stage aimed to separate the valid shoeprints from the complex backgrounds and conducting shoeprint registration and partition. Image preprocessing has the following steps:

(1) Shoeprint extraction: An image segmentation technique [24] is used to extract the shoeprint images from the complex backgrounds.
(2) Image registration: In shoeprint verification applications, accurate shoeprint alignment has a determinative effect, and a FFT-based registration algorithm [25] is used to align the shoeprint images.
(3) Shoeprint partition: A shoe partition model (SPM) is proposed to divide shoeprint image into different sections according to the structure of the foot and the relationship between shoe and foot. The SPM is to divide a shoeprint into different sections with a set of landmarks. Firstly, the contour of a shoeprint is represented by an average shape that is trained by using enough shoeprint images with various shapes. Secondly, three points (e.g., the front most point, rearmost point, and leftmost point) are marked interactively. Thirdly the other points of the contour, which are denoted with green dots as shown in Figure 5b, are estimated by using the interpolation method with the average shape and three points. Finally, the subsections are divided according to the predefined model. Each shoeprint is divided into toe section, sole section, instep section, heel section and back of heel section, and each section is further divided into several non-overlapped subsections for further analysis. The total number of subsections is 19.

### 3.2. Multi-Layer Feature Extraction

Most forensic experts use class, wear, and individual identifying characteristics to analyze shoeprints [1]. Class characteristics are usually caused through the manufacturing process and differ on shoe patterns. Wear characteristics are usually caused by natural erosion, and they reflect personal walking habits. Wear characteristics of shoes worn by different persons are different, even though the shoes are of the same patterns. Individual identifying characteristics are usually attributed to something randomly added to or taken away from the outsole, and they make the shoeprints unique. We propose a multi-layer feature extraction method to represent these characteristics of shoeprints on global layer, partial layer and individual identifying layer.

#### 3.2.1. Global Layer Feature Extraction

The global layer features that represent class characteristics are used to determine whether two shoeprints are of the same shoe patterns. Based on thousands of crime scene shoeprints, we found that patterns of the sole sections and the heel sections may be collected frequently in crime scenes, and they may determine the retrieval results in most cases. Thus, firstly, the shoeprint is divided into the bottom region and top region as shown in Figure 5a. Here, the ratio of the top region height to the bottom region height is set as 3 to 2, which is learned from training examples. Then the wavelet-Fourier–Mellin (WFM) transform coefficients of both the top and bottom regions are used to describe the class characteristics of the shoeprint [5]. For a given shoeprint image $u_{i}$, its global layer feature extraction process has two main steps as follows.

Step 1: The two corresponding regions $S_{\mathrm{top}}\left(i\right)$ and $S_{\mathrm{bottom}}\left(i\right)$ are decomposed by using Haar wavelet at a specified number of levels. We can acquire one approximation subband and three details at each level. The coefficients can be represented as:

(1) $$FW\left(S_{\mathrm{top}}\left(i\right)\right)\;=\;\left\{F_{{}^{W}}^{\left(S_{\mathrm{top}}\left(i\right)\right)}\left(l,h,v\right)|0\leq l\leq L,h,v=0,1\right\}$$

(2) $$FW\left(S_{\mathrm{bottom}}\left(i\right)\right)\;=\;\left\{F_{{}^{W}}^{\left(S_{\mathrm{bottom}}\left(i\right)\right)}\left(l,h,v\right)|0\leq l\leq L,h,v=0,1\right\}$$

where L denotes the maximum level. To avoid merging the useful neighbor patterns, L should meet the criterion: $2^{L-1}\leq D_{\min}$, where $D_{\min}$ represents the minimum distance between two neighbor patterns which can be specified interactively.

Step 2: Perform Fourier–Mellin transform on each band of wavelet coefficients, and then a band passed filter proposed in [25] is used to weaken the effect of connections between patterns and noises such as small holes and broken patterns. We use $FMW\left(S_{\mathrm{top}}\left(i\right)\right)$ and $FMW\left(S_{\mathrm{bottom}}\left(i\right)\right)$ to denote the global layer feature of the shoeprint $u_{i}$.

#### 3.2.2. Partial Layer Feature Extraction

**Figure 6:**
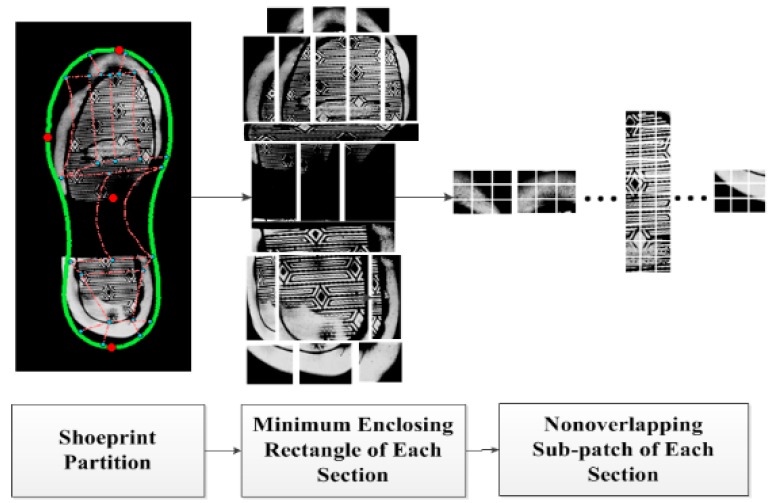
Illustration of the partial layer feature extraction method.

The partial layer features represent wear characteristics are used to determine whether two shoeprints have the same natural erosion caused by personal walking habits. Partial layer features are extracted in different subsections. The process of partial layer feature extraction are as follows, firstly the shoeprint is divided into nineteen subsections; secondly, the minimum enclosing rectangle region of each subsection is extracted and divided into non-overlapping patches; finally, the intensity distribution of each patch is used as the partial layer feature, which is represented as $F_{P}$. Figure 6 shows the process of partial layer feature extraction. 

According to human walking habits, the importance of subsections are different, so we defined subsection weights as shown in Table 1, and subsections that likely occur wear characteristics have larger weights.

#### 3.2.3. Individual Identifying Layer Feature Extraction

**Figure 7:**
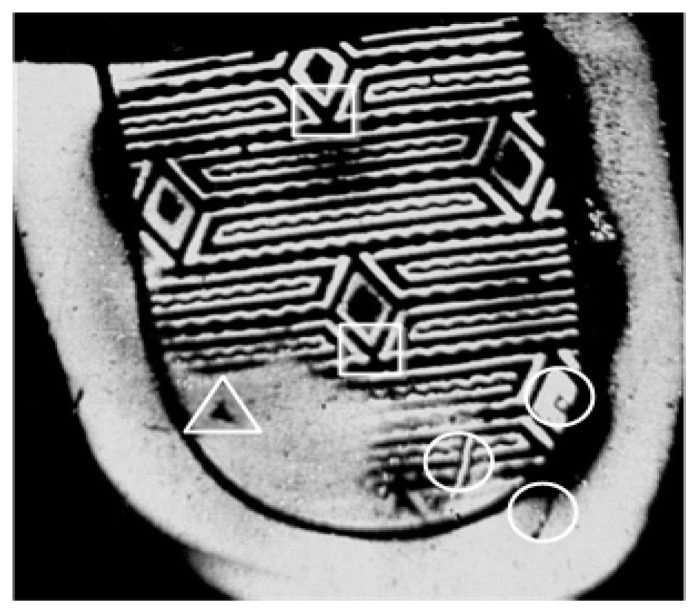
Examples of individual identifying characteristics. Areas labeled with circles are individual identifying characteristic with corner structure, and that labeled with triangles are individual identifying characteristics with blob-like structure. Areas labeled with rectangles are shoe patterns.

Individual identifying layer feature mainly detects the individual identifying characteristics caused by the material removed from the outsole or objects stuck on the outsole. An individual identifying layer feature may be represented as an interest point, line, or region on a shoeprint image. The word ‘individual’ here means that an individual feature should possess some very desirable criteria including nonrepeatability, that is, the individual feature cannot be detected in a shoeprint twice or two shoeprints left by different shoes. The individual identifying characteristics on a shoeprint mainly have two kinds of structures: the corner structure and the blob-like structure. Some samples of the two structures are shown in Figure 7.

To extract individual identifying layer features, the proposed method is based on two classes of interest point detectors. The two classes of feature points are described as follows:

The first class of feature points used in the proposed method is based on Harris corners. This class of feature points is used to detect the individual identifying layer features that have a corner structure. The individual identifying characteristic with a corner structure consists of corner points that can be the intersections of two edges, and these corners usually have conventional structures, such as L-corners, T-junctions, and Y-junctions. The Harris corner detector has a strong response to the intersections of two edges, and it detects corner points by using first order derivations of an image f as follows:

(3) $$A=\sum_{x}\sum_{y}w(x,y)\left[\begin{array}{ll} f_{x}^{2}(x,y) & f_{x}(x,y)f_{y}(x,y)\\ f_{x}(x,y)f_{y}(x,y) & f_{y}^{2}(x,y) \end{array}\right]$$

where $f_{x}$ and $f_{y}$ denote the first order derivative of f with respect to the x and y direction. Let w(x,y) represent the weighting function, and it is usually replaced by a Gaussian function g(x,y,σ) of a standard deviation σ. To detect the conventional corner structures described above, we perform the Harris corner detector on multiple scales, which detects Harris corners at different scales. A scaled image L with the scale parameter σ can be represented as:

(4) L(x,y,σ)=g(x,y,σ)×f(x,y)

The multiple scales Harris detector can be performed based on:

(5) $$A(x,y,\sigma_{i},\sigma_{d})=\sigma_{d}^{2}g(x,y,\sigma_{i})\left[\begin{array}{ll} L_{x}^{2}(x,y,\sigma_{d}) & L_{x}(x,y,\sigma_{d})L_{y}(x,y,\sigma_{d})\\ L_{x}(x,y,\sigma_{d})L_{y}(x,y,\sigma_{d}) & L_{y}^{2}(x,y,\sigma_{d}) \end{array}\right]$$

where $\sigma_{i}$ and $\sigma_{d}$ are the integration and differentiation scale, respectively [26,27].

We can compute the corner response of a point according to the advices in [28] as follows:

(6) $$R_{1}=\lambda_{1}\lambda_{2}-{\left(\lambda_{1}+\lambda_{2}\right)}^{2}=\det(A(x,y,\sigma_{i},\sigma_{d}))-k{\mathrm{trace}}^{2}(A(x,y,\sigma_{i},\sigma_{d}))$$

where $\lambda_{1}$ and $\lambda_{2}$ represent the eigenvalues of $A(x,y,\sigma_{i},\sigma_{d})$. k is a parameter that can be empirically set as 0.04.

As shown in Figure 7, individual identifying characteristics with the corner structure are always described by more than one corner point. We cluster these feature points in the position space, and choose center points of each cluster as the first class of candidate individual identifying characteristic points, and these points can be denoted as ${Β}_{1}=\{(x_{i},y_{i})|i=1,2,\ldots ,N_{1}\}$ where $\left(x_{i},y_{i}\right)$ denotes the position and $N_{1}$ is the number of the first class of candidate individual identifying characteristic points.

The second class of feature points in the proposed method is based on Hessian detector, and a Hessian detector can be performed by using the Hessian Matrix [27]. We use the Hessian detector to detect the individual identifying layer features with the blob-like structure, because the Hessian detector has a strong response to blobs. The Hessian Matrix can be represented as follows:

(7) $$A(x,y,\sigma_{d})=\left[\begin{array}{ll} L_{xx}(x,y,\sigma_{d}) & L_{xy}(x,y,\sigma_{d})\\ L_{xy}(x,y,\sigma_{d}) & L_{yy}(x,y,\sigma_{d}) \end{array}\right]$$

We can compute the Hessian corner response of a point by using the following formula:

(8) $$R_{2}=\det(A(x,y,\sigma_{d}))=L_{xx}L_{yy}-L_{xy}^{2}$$

The second class of candidate individual identifying characteristic points are denoted as ${Β}_{2}=\{(x_{i},y_{i})|i=1,\ldots ,N_{2}\}$, where $\left(x_{i},y_{i}\right)$ denotes the position and $N_{2}$ is the number of the second class of candidate individual identifying characteristic points.

**Figure 8:**
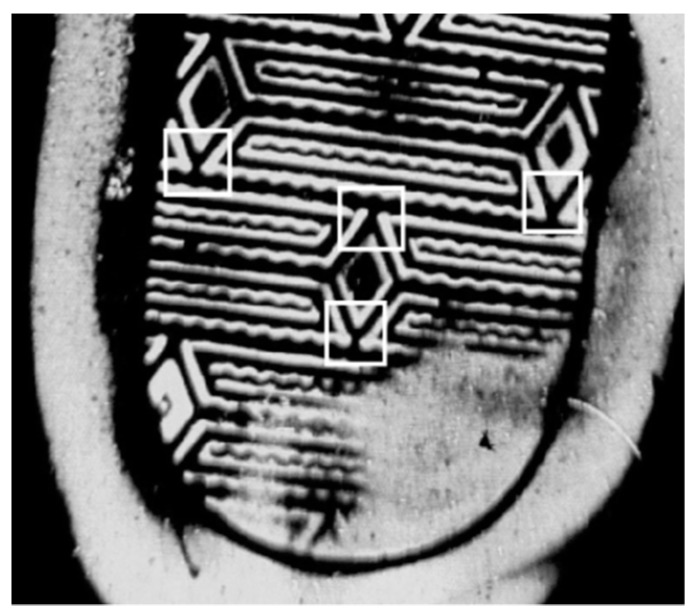
Periodic patterns that are labeled with white rectangles, and they are usually mistaken for individual identifying characteristic.

Uniqueness is the most visible trait that is used to distinguish individual identifying characteristics from shoe patterns. For a candidate individual identifying characteristic $(x_{i},y_{i})\in B$ and the patch centered on it, where $Β={Β}_{1}\cup {Β}_{2}=\{(x_{i},y_{i})|i=1,2,\ldots ,N\}$, and $N=N_{1}+N_{2}$. We use the intensity distribution of each patch centered on each point of Β to describe these points. Individual identifying characteristics are usually produced on the outsole randomly, which means that the individual identifying characteristics must be unique in a local region. In the process of individual identifying characteristic detection, periodic patterns are usually mistaken for individual identifying characteristic, as shown in Figure 8. We tell whether a point $\left(x_{i},y_{i}\right)$ has the same pattern as point $\left(x_{j},y_{j}\right)$ by calculating the normalized cross-correlation of the Fourier feature of the regions centering at these points:

(9) $$s\left(f\left(R_{i}\right),f\left(R_{j}\right)\right)=\frac{\left(f\left(R_{i}\right)-\mu_{f\left(R_{i}\right)}\right)\left(f\left(R_{j}\right)-\mu_{f\left(R_{j}\right)}\right)}{\sigma_{f\left(R_{i}\right)}\sigma_{f\left(R_{j}\right)}}$$

where $f\left(R_{i}\right)$ denote the Fourier feature of the patch centered at point $\left(x_{i},y_{i}\right)$. Then we delete the similar points which have the same patterns. By this way, we can get a refined individual identifying characteristic point set ${Β}_{p}$, and we use the intensity distribution of each patch centered on each point of ${Β}_{p}$ as individual identifying layer features, which is represented as $F_{I}$.

### 3.3. Multi-Layer Feature Matching

Two matching techniques are introduced in the work presented in this paper, which are the wavelet-Fourier–Mellin transform and similarity estimation (WFSE) method [5] and Ciratefi [29]. Matching using WFSE method computes the wavelet-Fourier–Mellin feature distances between the probe shoeprint and questioned shoeprint. The second matching technique matches a patch in the probe shoeprint against patches in the questioned shoeprint and computes the matching score with the best matching patch.

For the global layer feature of a pair of shoeprints U, similarities of the top region and the bottom region are computed according to the 2D correlation coefficient, respectively, and the similarity value of class characteristics $P_{G}(U)$ is computed by their correlation coefficient of global layer features. It is a weighted sum of correlation coefficients of both $FMW\left(S_{\mathrm{top}}\left(i\right)\right)$ and $FMW\left(S_{\mathrm{bottom}}\left(i\right)\right)$. Please refer to [5] for details.

**Figure 9:**
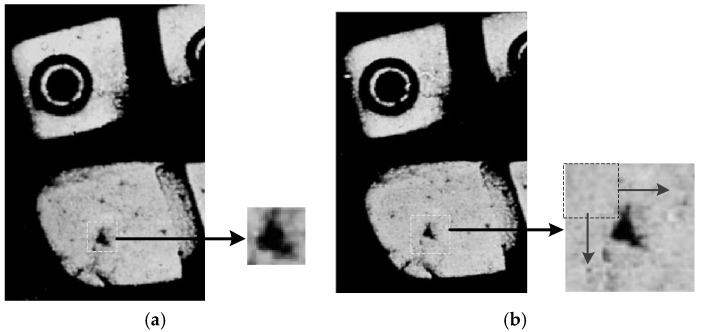
Illustration of template matching method. (**a**) The probe patch; (**b**) The extension patch in the questioned shoeprint.

With regard to the partial layer feature and individual identifying layer feature, we used a template matching method named Ciratefi [29] to find its best matching patch within a searching window of the questioned shoeprint. Figure 9 shows an example of the template matching method. An individual identifying layer feature F is extracted and encircled by the white dotted box as shown in Figure 9a, and patch A is an extension patch and locates at the same position in the questioned shoeprint as shown in Figure 9b, we aimed at finding the most similar patch with feature F in patch A, and assigned the maximum similarity value as the matching score S(F), which represents the similarity value of two shoeprints on feature F. The method compares feature F with every patch in a sliding window manner from patch A. The template matching method integrates three cascaded filters to yield a satisfactory result. The three filters are circular sampling filter, radial sampling filter, and template matching filter. The former two filters are used to eliminate candidate patches that have no opportunity to match with the probe template F, and the last filter is utilized to find the best matching patch and compute the matching score.

The process of the template matching method is detailed as follows. A circular sampling filter projects the images A and F on a set of circular rings, and uses these projections to detect the first level candidate patches and their corresponding matching scales. Given the template F, a scaled image $F_{i}$ with the scale parameter $\sigma_{i}(i=1,2,\ldots ,n)$ can be represented by using Equation (4). Then, each scaled image $F_{i}$ is circularly sampled in a set of circle rings situated at radius $r_{k}(k=1,2,\ldots ,l)$ from the center pixel (x,y):

(10) $$C_{F}(i,k)=\frac{1}{2\pi r_{k}}\sum_{\theta =0}^{2\pi }F_{i}(x+r_{k}\cos\theta ,y+r_{k}\sin\theta )$$

With regard to the image A, the circular projections for each $\left(x_{0},y_{0}\right)$ in A can be denoted as:

(11) $$C_{A}(x_{0},y_{0},k)=\frac{1}{2\pi r_{k}}\sum_{\theta =0}^{2\pi }A(x_{0}+r_{k}\cos\theta ,y_{0}+r_{k}\sin\theta ),(x_{0},y_{0})\in A$$

The correlation coefficient of $C_{F}$ and $C_{A}$ is used to detect the best matching scale for each pixel $\left(x_{0},y_{0}\right)$ in A, which can be represented as:

(12) $$X(x_{0},y_{0})=\max\left(\frac{\left(C_{F}(i)-\bar{C_{F}(i)})(C_{A}(x_{0},y_{0})-\bar{C_{A}(x_{0},y_{0})}\right)}{\left\|C_{F}(i)-\bar{C_{F}(i)}\right\|\left\|\left(C_{A}(x_{0},y_{0})-\bar{C_{A}(x_{0},y_{0})}\right)\right\|}\right)$$

A candidate pixel $\left(x_{0},y_{0}\right)$ in A can be detected as the first grade candidate pixel if $X(x_{0},y_{0})>t_{1}$, where $t_{1}$ denotes the threshold. The corresponding best matching scale of the first-grade candidate pixel $\left(x_{0},y_{0}\right)$ can be obtained as follows:

(13) $$G_{1}(x_{0},y_{0})=\underset{i}{\arg\max}\left(\frac{\left(C_{F}(i)-\bar{C_{F}(i)})(C_{A}(x_{0},y_{0})-\bar{C_{A}(x_{0},y_{0})}\right)}{\left\|C_{F}(i)-\bar{C_{F}(i)}\right\|\left\|\left(C_{A}(x_{0},y_{0})-\bar{C_{A}(x_{0},y_{0})}\right)\right\|}\right)$$

A radial sampling filter projects the images A and $F_{i}$ on a set of radial lines, and uses these projections to eliminate the first level candidate that have no opportunity to match the probe template $F_{i}$. Radial sampling filter can obtain the second-grade candidate pixels by eliminating some of the first grade candidate pixels; meanwhile, it can also obtain their corresponding matching orientations. Given a first-grade candidate pixel $\left(x_{0},y_{0}\right)$ and its corresponding matching scale $\sigma_{i}(i=G_{1}(x_{0},y_{0}))$, the scaled template image $F_{i}$ with the scale parameter $s_{i}$ can be represented by using Equation (4). The scaled image $F_{i}$ is radially sampled on the radial line situated at radius $r_{l}$ from the center pixel (x,y) and inclination $\theta_{j}$:

(14) $$R_{F}(j)=\frac{1}{r_{l}}\sum_{t=0}^{r_{l}}F_{i}(x+r_{l}\cos\theta_{j},y+r_{l}\sin\theta_{j}),0\leq j<m$$

For each first-grade candidate pixel $\left(x_{0},y_{0}\right)$, the radial projections can be denoted as:

(15) $$R_{A}(x_{0},y_{0},j)=\frac{1}{r_{l}}\sum_{t=0}^{r_{l}}A(x_{0}+r_{l}\cos\theta_{j},y_{0}+r_{l}\sin\theta_{j}),0\leq j<m$$

The correlation coefficient of $R_{F}$ and $R_{A}$ is used to detect the best matching scale for each pixel $\left(x_{0},y_{0}\right)$ in A, which can be represented as:

(16) $$Y(x_{0},y_{0})=\max\left(\frac{\left(R_{F}(i)-\bar{{\mathrm{shift}}_{j}R_{F}(i)})(R_{A}(x_{0},y_{0})-\bar{{\mathrm{shift}}_{j}R_{A}(x_{0},y_{0})}\right)}{\left\|R_{F}(i)-\bar{{\mathrm{shift}}_{j}R_{F}(i)}\right\|\left\|\left(R_{A}(x_{0},y_{0})-\bar{{\mathrm{shift}}_{j}R_{A}(x_{0},y_{0})}\right)\right\|}\right)$$

where “shift” means circular shifting j positions of the argument vector. The first grade candidate pixel $\left(x_{0},y_{0}\right)$ in A can be reserved and updated as the second grade candidate pixel if $Y(x_{0},y_{0})>t_{2}$, and $t_{2}$ denotes the threshold. The corresponding best matching orientation of the second-grade candidate pixel $\left(x_{0},y_{0}\right)$ can be obtained as follows:

(17) $$G_{2}(x_{0},y_{0})=\underset{i}{\arg\max}\left(\frac{\left(R_{F}(i)-\bar{{\mathrm{shift}}_{j}R_{F}(i)})(R_{A}(x_{0},y_{0})-\bar{{\mathrm{shift}}_{j}R_{A}(x_{0},y_{0})}\right)}{\left\|R_{F}(i)-\bar{{\mathrm{shift}}_{j}R_{F}(i)}\right\|\left\|\left(R_{A}(x_{0},y_{0})-\bar{{\mathrm{shift}}_{j}R_{A}(x_{0},y_{0})}\right)\right\|}\right)$$

The template matching filter computes the similarity between the patch X centered at each second grade candidate pixel $\left(x_{0},y_{0}\right)$ and the scaled and rotated template $F_{i}$ that use the best matching scale $\sigma_{i}(i=G_{1}(x_{0},y_{0}))$ and the best matching angle $\theta_{j}(j=G_{2}(x_{0},y_{0}))$, and the structural similarity (SSIM) method proposed in [30] was used to evaluate the similarity as follows:

(18) $$SSIM(X,F_{i})={\left[\frac{2\mu_{X}\mu_{F}+C_{1}}{\mu_{X}^{2}+\mu_{F}^{2}+C_{1}}\right]}^{\alpha }{\left[\frac{2\sigma_{X}\sigma_{F}+C_{2}}{\sigma_{X}^{2}+\sigma_{F}^{2}+C_{2}}\right]}^{\beta }{\left[\frac{\sigma_{XF}+C_{3}}{\sigma_{X}\sigma_{F}+C_{3}}\right]}^{\gamma }$$

where $\mu_{X}$ denotes the mean of X, $\sigma_{X}$ denotes the standard deviation of X, and $\sigma_{XF}$ denotes the covariance of X and $F_{i}$. The constants $C_{1}$,$C_{2}$, and $C_{3}$ are used to avoid the denominators being close to zeroes. α, β, and γ are parameters which are used to adjust the importance of the corresponding components.

The second grade candidate pixel $\left(x_{0},y_{0}\right)$ with the highest structural similarity is chosen, and the template is considered to be found at pixel $\left(x_{0},y_{0}\right)$, and the highest structural similarity is set as the feature similarity of this patch.

The similarity values of partial and individual identifying layer features between two shoeprints are calculated by using the Ciratefi method. The similarity values of two-layer features in subsection $s_{i}$ are computed as follows:

(19) $$P_{P}\left(s_{i}\right)=\sum_{F_{P,m}\in s_{i}}S\left(F_{P,m}\right)/N_{P}\left(s_{i}\right)\left(i=1,2,\ldots ,19\right)$$

(20) $$P_{I}\left(s_{i}\right)=\sum_{F_{I,n}\in s_{i}}S\left(F_{I,n}\right)/N_{I}\left(s_{i}\right)\left(i=1,2,\ldots ,19\right)$$

where $F_{P,m}$ and $F_{I,n}$ denote the mth partial layer patch and nth individual identifying layer patch in the subsection $s_{i}$, respectively, and $S\left(F_{P,m}\right)$ and $S\left(F_{I,n}\right)$ represent the similarity value of two shoeprints on feature $F_{P,m}$ and $F_{I,n}$. $N_{P}\left(s_{i}\right)$ and $N_{I}\left(s_{i}\right)$ denote the number of partial layer features and individual identifying layer features in $s_{i}$, respectively. $P_{P}\left(U\right)=\left\{P_{P}(s_{i})|i=1,2,\cdots ,19\right\}$ and $P_{I}\left(U\right)=\left\{P_{I}(s_{i})|i=1,2,\cdots ,19\right\}$ represent partial and individual identifying characteristics similarity value set of two shoeprints in nineteen subsections.

### 3.4. Decision-Making

For fusing the three layer features at the score level, the total similarity score $p\in {\mathbb{R}}_{+}$ is used to denote the possibility that two shoeprints could have been made by the same shoe. For a pair of shoeprints U, their total similarity score p is defined as a function of the similarity values of global, partial, and individual identifying layer features, which can be expressed as

(21) $$p=f\left(P_{G},P_{P},P_{I},P_{W}\right)=\mathrm{sgn}\left(P_{G}-T_{G}\right)P_{\mathrm{PI}}$$

where sgn(•) is a sign function. $T_{G}$ denotes a threshold that is used to determine whether the two shoeprints have the same class characteristics. $P_{\mathrm{PI}}$ represents the combined feature score of two shoeprints on both partial and individual identifying layer features, which is mathematically defined as follows:

(22) $$P_{\mathrm{PI}}=\frac{1}{19}\sum_{i=1}^{19}P_{W}\left(s_{i}\right)P_{P}\left(s_{i}\right)P_{I}\left(s_{i}\right)$$

where $P_{W}(s_{i})$ is the weight value of subsection $s_{i}$, and $P_{W}(s_{i})=1-R(s_{i})/\sum_{i}R(s_{i})$, where $R(s_{i})$ represents the weight order, which are listed in Table 1.

For a pair of shoeprints, if p≤0, then we can make a conclusion that the two shoeprints may have different patterns; if p is greater than a positive predefined threshold $T_{T}$, then we conclude that the two shoeprints could be left by the same shoe, otherwise they could not be left by the same shoe. The decision rule can be described as follows:

(23) $$V\left(p\right)=\{\begin{array}{cc} 1 & p>T_{T}\\ 0 & 0\leq p\leq T_{T}\\ 0 & p<0 \end{array}$$

where V(p)=1 indicates the two shoeprints are from the same shoe, and V(p)=0 denotes the two shoeprints are from different shoes.

Accord to Equations (21) and (23), two thresholds $T_{G}$ and $T_{T}$ are needed to be pre-trained to tell whether two shoeprint are from the same shoe. We used the decision cost function (DCF) [31] to choose these two thresholds, that is to say

(24) $$\left[{\hat{T}}_{G},{\hat{T}}_{T}\right]=\arg\underset{T_{G},T_{T}}{\min}\left(FAR(T_{G},T_{T})+FRR(T_{G},T_{T})\right)$$

where $FAR(T_{G},T_{T})$ and $FRR(T_{G},T_{T})$ denote the false acceptance rate and the false rejection rate at the threshold $T_{G}$ and $T_{T}$, which can be calculated with Equations (25) and (26).

The proposed multi-layer-based shoeprint verification method is summarized in Algorithm 1.

**Algorithm 1.** Shoeprint Image Verification Algorithm**Input:** A pair of shoeprints U. **Output:** The total similarity score p and the verification result. **1.** Image preprocessing. **2.** Feature extraction. Extract partial layer feature, and individual identifying layer feature, respectively. **3.** Feature matching. **4.** Calculate similarity of global layer feature. **5. For** r = 1, 2, …, 19 **6.** Calculate similarity of partial layer feature with Equation (19). **7.** Calculate similarity of individual identifying layer feature with Equation (20). **8. end For**
**9.** Calculate total similarity score with Equation (21). **10.** Judgment. Output verification result, identical or non-identical.

## 4. Experiments

In this section, we evaluate the proposed method by conducting shoeprint verification experiments on the real crime scene shoeprint image database and the suspect shoeprint image database. The false rejection rate (FRR), the false acceptance rate (FAR), and the equal error rate (EER) are used to evaluate the proposed method. The following subsections detail the experiment configuration, performance evaluation, analysis and discussion.

### 4.1. Experiment Configuration

In the experiments, we automatically aligned each pair of shoeprints. Thus, the main differences between the shoeprints are of a structural nature (i.e., the class characteristics, wear characteristics, and individual identifying characteristics). The registration make it possible to compare our method with some robust methods that are not invariant to rigid transformations, e.g., the histogram of oriented gradients (HOG) method [32] and the normalized cross correlation method (NCC). We also compared some RST invariant methods, e.g., the SURF algorithm [33] with the proposed method.

#### 4.1.1. Dataset

The dataset consisted of one testing set and one training set. The definitions and descriptions of the two kinds of datasets are detailed as follows.

(i) Testing set: A testing set is a collection of shoeprint images that need to be verified. The testing set contained 1200 pairs of reference shoeprints in the MUES-SV1KR2R dataset and 256 pairs of crime scene shoeprints in the MUES-SV2HS2S dataset.
(ii) Training set: The training set is a collection of shoeprint images used to train the thresholds. The training set consisted of 300 pairs of reference shoeprint images and 100 pairs of crime scene shoeprint images. One hundred pairs of reference shoeprint images were from the same shoes. Twenty-five pairs of crime scene shoeprints are from the same shoes, and fifty pairs of shoe prints were not of the same class characteristics. Then accord to Equation (24), the optimal $T_{G}$ and $T_{T}$ can be achieved by operating shoeprint verification on the training set.

#### 4.1.2. Evaluation Metric

In our experiments, we used the false acceptance rate (FAR), the false rejection rate (FRR), and the equal error rate (EER) to evaluate and characterize the proposed shoeprint verification system. These evaluation methods are always used to evaluate the performance of biometric verification, such as the finger vein verification [34,35] and palm vein verification [36].

The false acceptance rate means that deciding a claimed identity is a legitimate one while in reality it is an imposter. The FAR can be expressed as follows:

(25) $$FAR=\frac{N_{\mathrm{FA}}}{N_{\mathrm{IA}}}\times 100\%$$

where $N_{\mathrm{FA}}$ and $N_{\mathrm{IA}}$ denote the number of false acceptances and the number of imposter attempts, respectively.

The false rejection rate means that deciding that a claimed identity is a not legitimate one while in reality it is the genuine one. The FRR can be expressed as follows:

(26) $$FRR=\frac{N_{\mathrm{FR}}}{N_{\mathrm{TA}}}\times 100\%$$

where $N_{\mathrm{FR}}$ and $N_{\mathrm{TA}}$ denote the number of failed rejections and the number of target access, respectively.

The equal error rate (EER) represents the point where the false acceptance rate and the false rejection rate are equal, and a good system should keep this value as small as possible.

### 4.2. Performance Evaluation

In this section, we conducted two kinds of experiments to evaluate the performance of the proposed method. The first kind of experiment was to verify the performance of the proposed shoeprint verification method. The second kind of experiments was to compare the proposed method with some conventional methods.

#### 4.2.1. Performance Evaluation of the Proposed Method

**Figure 10:**
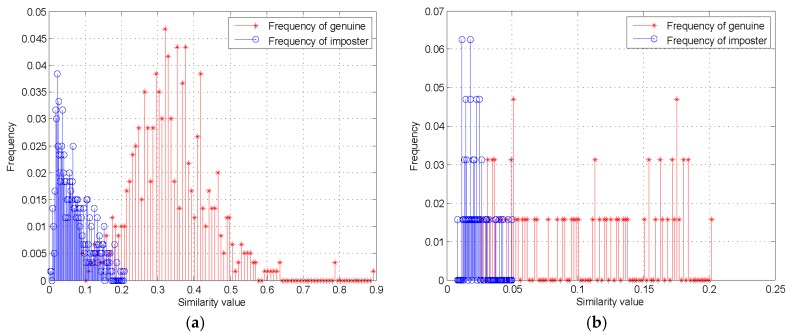
Genuine and imposter distributions of the proposed method on the datasets. (**a**) Results on the MUES-SV1KR2R dataset; (**b**) results on the MUES-SV2HS2S dataset.

We evaluated the performance of the proposed method on two shoeprint datasets, and the datasets consisted of genuine and imposter pairs that included intra-class and inter-class matches. In this work, intra-class matching was defined as matching between a pair of shoeprint samples that were made by the same shoe, and inter-class matching was defined as matching between a pair of shoeprint samples that were left by different shoes. The experimental results are shown as follows, and Figure 10 shows the histograms of the similarity value for genuine and imposter shoeprint samples in MUES-SV1KR2R and MUES-SV2HS2S dataset, respectively. The left blue lines show the frequency distribution for inter-class similarity value, while the right red lines show the frequency distribution for intra-class similarity value. The results show that the frequency distribution of similarity value of inter-class are significantly lower than that of intra-class, which implies that the similarity value of the multiple-layer features for shoeprint samples left by the same shoe is significantly higher than the similarity value for shoeprint samples left by different shoe. The results also show that it is possible to tell whether two shoeprints are left by the same shoe.

#### 4.2.2. Comparison and Discussion

**Figure 11:**
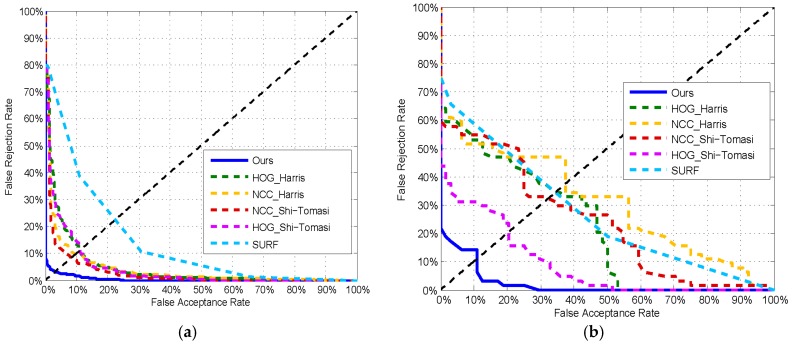
The receiver operating characteristics (ROC) curves of different methods on the datasets. (**a**) ROC curves on the MUES-SV1KR2R dataset; (**b**) ROC curves on the MUES-SV2HS2S dataset.

We also used the equal error rate to evaluate the performance of the methods on two datasets by making trade-offs between the false acceptance rate and the false rejection rate. The performance of the methods is shown in the Figure 11, where the equal error rates of the proposed method were 3.2% and 10.9% on the MUES-SV1KR2R and MUES-SV2HS2S, respectively.

To our best knowledge, there is no automatic shoeprint verification algorithm reported up to now, so we could not compare the proposed algorithm with the state-of-the-art methods. To further test the effectiveness of the proposed method, we conducted comparisons with some existing conventional interest point-based methods. To conduct these comparisons, we implemented the conventional methods based on the corresponding literatures. In the experiments, we mainly compared our method with some interest point detections, such as the Harris [28] and Shi-Tomasi [37] corner detectors. We integrated the normalized cross correlation method (NCC) and the histograms of oriented gradients method (HOG) with the interest point detections. The patch centered on each interest point was used to describe the point, and HOG and NCC methods were used to describe the patch and match shoeprints. We also compared some RST invariant methods with the proposed method, e.g., the SURF algorithm [33].

The compared methods were all implemented with MATLAB codes. The patch size was fixed in all experiments. To ensure a fair comparison, all of the shoeprints in the datasets were aligned. The receiver operating characteristics (ROC) curve on the MUES-SV1KR2R dataset are illustrated in Figure 11a, and the corresponding results are listed in Table 2. The results show that the NCC method and HOG method perform nearly equally for all interest points, respectively, and the NCC method can perform better than the HOG method on average. The reason may be that shoeprint registration is performed by using a FFT-based registration algorithm [25], and there are variations on transformation between shoeprints. NCC methods can be robust to transformational deviations. If there are not large variations on transformation between shoeprint, the HOG method may surpass the NCC method, because HOG methods take into consideration local structural information and can be robust to small transformational deviations by pooling local structural information into histograms. The results in general show that all of the methods perform well on MUES-SV1KR2R dataset. Our approach performs significantly better compared to the compared methods.

The receiver operating characteristics (ROC) curve on the MUES-SV2HS2S dataset are illustrated in Figure 11b, and the corresponding results are listed in Table 3. The results show that HOG method can perform better than the NCC method. The reason may be that shoeprints in the MUES-SV2HS2S dataset are always of low quality and shoeprints made by the same shoe are not exactly the same due to the varying imaging conditions. HOG methods take into consideration local structural information and can be robust to small transformational deviations by pooling local structural information into histograms. The results also show that the EER of all of the compared methods increase a lot, compared with shoeprint verification on MUES-SV1KR2R dataset. The main reason may be that shoeprint images in the MUES-SV2HS2S dataset are of low quality and with complicated backgrounds, and it results in that the interest points always lying on the shoeprint impression boundary and background. Our approach performes significantly better compared to the other feature detector and descriptor combinations. The results are summarized in Table 1. Our method also performes better than on the Harris, Shi-Tomasi, and SURF interest point method. That is mainly because (i) the multi-layer features used in our method use global and local information to analyze shoeprints and integrate their strengths to yield more satisfactory verification results; and (ii) we analyze and estimate the similarity of the local structural information by using the SSIM method.

**Figure 12:**
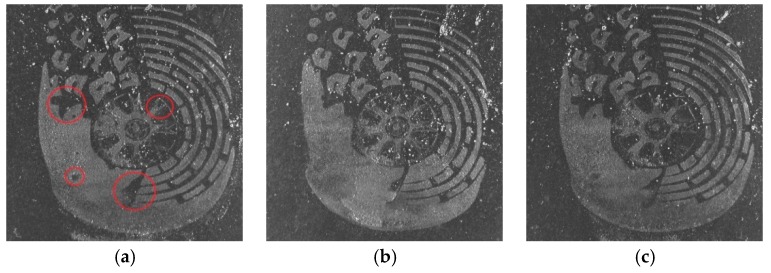
Samples of the probe shoeprint and two questioned shoeprints. (**a**) The probe shoeprint sample; (**b**) the questioned shoeprint sample that is made by different shoes with (**a**); (**c**) the questioned shoeprint sample that is made by the same shoe with (**a**).

**Figure 13:**
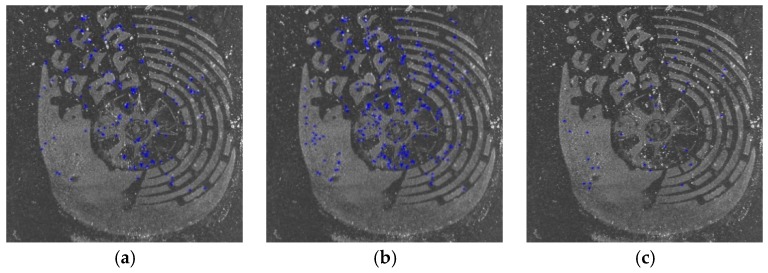

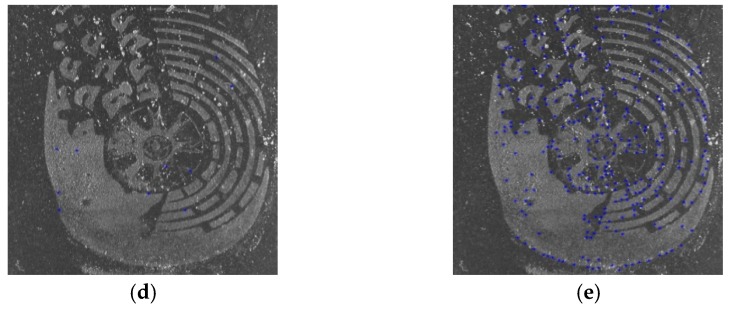
The results of our method and the compared methods on shoeprint made by different shoes. The unmatched points are labeled with blue ‘*’s. The more points are labeled, which should include red circled points in Figure 12a at least, the better the algorithm performs. (**a**) The results of Harris_HOG; (**b**) the results of Harris_NCC; (**c**) The results of Shitomasi_NCC; (**d**) the results of Shitomasi_HOG; (**e**) the results of our method.

**Figure 14:**
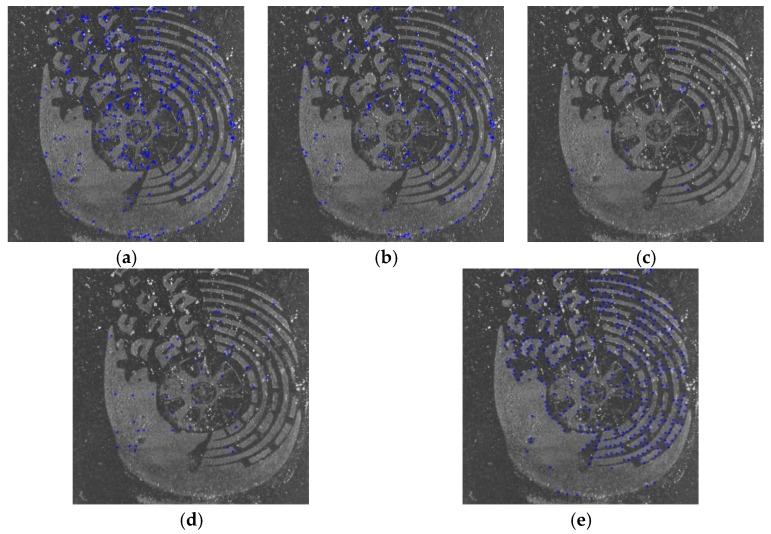
The results of the proposed and the compared methods on shoeprints made by the same shoe. The matched points are labeled with blue ‘*’s. The more points are labeled, which should include red circled points in Figure 12a at least, the better the algorithm performs. (**a**) The results of Harris_HOG; (**b**) the results of Harris_NCC; (**c**) the results of Shitomasi_NCC; (**d**) the results of Shitomasi_HOG; (**e**) the results of our method.

Figure 12 shows three samples of shoeprint in the MUES-SV2HS2S dataset. The shoeprints in Figure 12 have the same shoe pattern, and the shoeprint shown in Figure 12b is left by a different shoe from Figure 12a,c. The main individual identifying characteristics are labeled with red circles as shown in Figure 12a. Figure 13 and Figure 14 provide a visual illustration of the shoeprint verification results of the proposed method and the compared methods. We extract individual identifying characteristics on Figure 12a and compare these extracted characteristics at the same position in Figure 12b,c. The results between Figure 12a,b are shown in Figure 13, and the results between Figure 12a,c are shown in Figure 14. In Figure 13, the unmatched points are labeled with blue ‘*’ s. The unmatched points denote that the two shoeprints in the point location were different. The matched points are labeled with blue ‘*’s in Figure 14. The results show that our approach can tell not only the differences between two shoeprint images but also the characteristics in common. The results show that the proposed method outperformed the compared methods.

Finally, we compare the time-consumption of the methods stated previously. This kind of experiments is conducted on a Windows 64-bit system with a 3.3-GHz CPU and 8-GB RAM. The time consumptions of each method on the two datasets are shown in Table 2 and Table 3. Experimental results show that the proposed method required 280.6 s and 293.3 s of computation time for shoeprint verification on the two datasets. Although the proposed method requires more time to verify shoeprints than the compared methods, the performance of the proposed method surpasses the compared methods. Compared with the methods stated above, the proposed method matches not only the individual identifying layer feature, but also the global layer and partial layer feature, so it requires more computation. It is also for that more time is spent on the calling the Ciratefi method, because the Ciratefi method needs a lot of computations. In future works, we would like to reduce computation time by introducing parallel computing technology and optimizing the program code.

## 5. Conclusions

In this work, we have proposed a multi-layer feature-based method to conduct shoeprint verification automatically. Multi-layer features were extracted to compute the similarity values of two shoeprints on each layer, and then a total similarity score was computed based on these similarity values and a conclusion was made by a piecewise function of total similarity score. Comparative experiments with state-of-the-art methods were conducted, and the experimental results showed that: (1) our method had a better performance than the SURF method as well as some other state-of-the-art methods; and (2) the higher the quality of the shoeprint images were, the lower the EER of the methods can be achieved. In conclusion, the EERs on the two shoeprint image databases, which were 3.2% and 10.9%, demonstrate that our method is an effective shoeprint verification method, especially for images derived by using the shoeprint scanners. Our next work is to design a much more effective classifier to improve the verification algorithm, and we will also focus on how to improve the performance on complex backgrounds.

## Figures and Tables

**Table 1:** Weight order of each subsection in a shoeprint.

Subsection Index	Section of Shoeprint	Weight Order
1, 2, 3	Toe	3
4, 5, 6, 7, 8, 9	Sole	1
13, 14, 15, 16	Heel	2
17, 18, 19	Back of Heel	4
10, 11, 12	Instep	5

**Table 2:** Summary of the experiments in terms of shoeprint verification performance on the MUES-SV1KR2R dataset measured by the equal error rate (EER).

Method for Shoeprint Verification	Performance (EER), %	Computation Time, s
Harris_HOG	11.1	2.1
Harris_NCC	9.8	3.1
Shi-Tomasi_HOG	11.2	9.7
Shi-Tomasi_NCC	8.5	10.0
SURF	22.4	14.7
**Ours**	**3.2**	**280.6**

**Table 3:** Summary of the experiments in terms of shoeprint verification performance on the MUES-SV2HS2S dataset measured by the EER.

Method for Shoeprint Verification	Performance (EER), %	Computation Time, s
Harris_HOG	34.4	2.1
Harris_NCC	37.5	3.2
Shi-Tomasi_HOG	20.3	10.4
Shi-Tomasi_NCC	31.3	11.0
SURF	34.3	15.2
**Ours**	**10.9**	**293.3**
